# SSE: a nucleotide and amino acid sequence analysis platform

**DOI:** 10.1186/1756-0500-5-50

**Published:** 2012-01-20

**Authors:** Peter Simmonds

**Affiliations:** 1 Centre for Immunity, Infection and Evolution, University of Edinburgh, Ashworth Laboratories, Kings Buildings, West Main Road, Edinburgh EH9 3JT, UK

## Abstract

**Background:**

There is an increasing need to develop bioinformatic tools to organise and analyse the rapidly growing amount of nucleotide and amino acid sequence data in organisms ranging from viruses to eukaryotes.

**Finding:**

A simple sequence editor (SSE) was developed to create an integrated environment where sequences can be aligned, annotated, classified and directly analysed by a number of built-in bioinformatic programs. SSE incorporates a sequence editor for the creation of sequence alignments, a process assisted by integrated CLUSTAL/MUSCLE alignment programs and automated removal of indels. Sequences can be fully annotated and classified into groups and annotated of sequences and sequence groups and access to analytical programs that analyse diversity, recombination and RNA secondary structure. Methods for analysing sequence diversity include measures of divergence and evolutionary distances, identity plots to detect regions of nucleotide or amino acid homology, reconstruction of sequence changes, mono-, di- and higher order nucleotide compositional biases and codon usage.

Association Index calculations, GroupScans, Bootscanning and TreeOrder scans perform phylogenetic analyses that reconcile group membership with tree branching orders and provide powerful methods for examining segregation of alleles and detection of recombination events. Phylogeny changes across alignments and scoring of branching order differences between trees using the Robinson-Fould algorithm allow effective visualisation of the sites of recombination events.

RNA secondary and tertiary structures play important roles in gene expression and RNA virus replication. For the latter, persistence of infection is additionally associated with pervasive RNA secondary structure throughout viral genomic RNA that modulates interactions with innate cell defences. SSE provides several programs to scan alignments for RNA secondary structure through folding energy thermodynamic calculations and phylogenetic methods (detection of co-variant changes, and structure conservation between divergent sequences). These analyses complement methods based on detection of sequence constraints, such as suppression of synonymous site variability.

For each program, results can be plotted in real time during analysis through an integrated graphics package, providing publication quality graphs. Results can be also directed to tabulated datafiles for import into spreadsheet or database programs for further analysis.

**Conclusions:**

SSE combines sequence editor functions with analytical tools in a comprehensive and user-friendly package that assists considerably in bioinformatic and evolution research.

## Background

The bioinformatic analysis of nucleotide (and translated) amino acid sequence data is undergoing a revolution through recent developments in data acquisition and sequencing methodologies. Analysis of the organisation, gene complement and regulatory elements in large genomes (such as from prokaryotes and eukaryotes) and the diversity of populations in viral and increasingly in larger genomes places increasing demands on the bioinformatic tools used to manage such data. Effective alignment, sequence annotation, comparison and genetic analysis require platforms able to handle large file sizes and efficiently process and analyse large nucleotide sequence datasets. There is additionally the analytical challenge, to investigate diversity, composition and phylogenies in large datasets. Analysis of naturally occurring diversity provides large amounts of information on the evolutionary processes, constraints and mechanisms of sequence change and on the existence of quantitative and qualitative differences in selection pressures exerted on virus, bacteria and eukaryotic genomes.

To address many of these issues, a simple sequence editor (SSE) has been developed as a general purpose program to organise, annotate and align nucleotide and amino acid sequence datasets, and provide an integrated platform for a range of analytic methods for investigation of diversity, compositional selection pressures and recombination. The program represents a major upgrade from a previous software package, Simmonics (versions 1.0-1.6) with substantial enhancement of functionality through its Windows interface and additional research tools. SSE, with its effective visualisation of analytical results provides the non-specialist user a gentler introduction into bioinformatic and evolutionary analysis than currently provided by R, BioPerl and associated scripting languages.

## Finding

### Implementation

SSE is installed through a standard installer package (approximately 13 MB) from http://www.virus-evolution.org/Downloads/Software. It subsequently runs as a standalone program on personal computers (Windows XP, NT, Vista or Windows 7, Macs running WINE or Virtualisation environments [VMware or parallels] with Windows). Functionality of the package is enhanced by pre-installation of PHYLIP (version 3.5 onwards) from http://evolution.genetics.washington.edu/phylip.html and UNAFOLD from http://mfold.rna.albany.edu/?q=DINAMelt/software. These installed packages are identified on first program run of SSE or can be searched or specified after installation. SSE additionally installs the program DPlot that plots program output in real time and the alignment programs CLUSTALW [1] and MUSCLE [2] that run within the package for automated nucleotide and amino acid sequence alignment.

SSE imports and exports all standard nucleotide and amino acid sequence formats (*eg*. FASTA, PIR, PHYLIP, MEGA, CLUSTAL, NEXUS) as well as importing standard database output (GenBank and EMBL) from which sequence annotations are assimilated and optionally stored. Sequence alignments are stored by default in a SSE database format that retains sequence editor environment selections and sequence groupings/descriptions and other file annotations.

**Figure 1:**
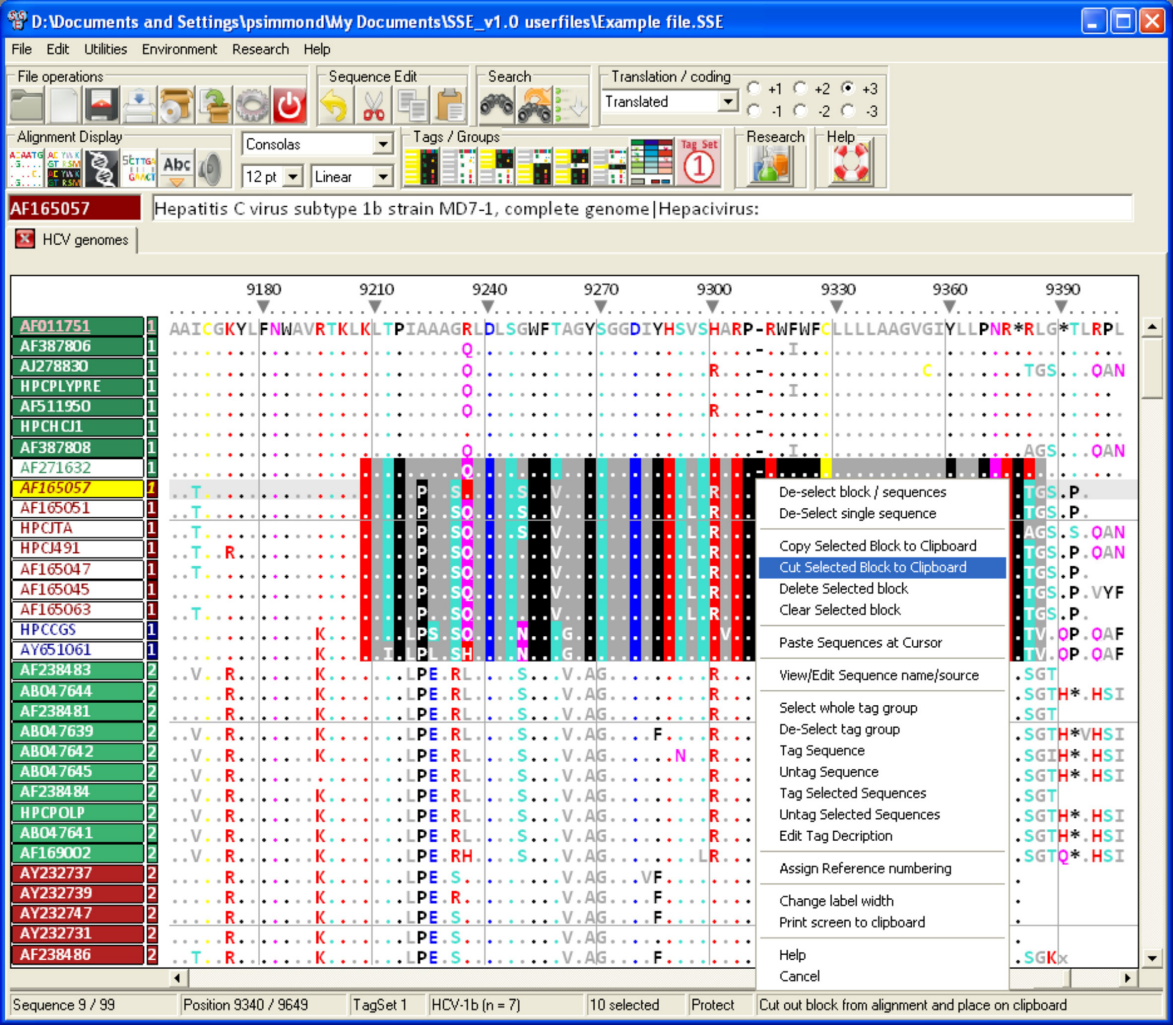
**The basic editor screen for direct alignment editing, GUI controls, context and window menus**. The main editing screen showing translated nucleotide sequences of hepatitis C virus (HCV) and options available on a context menu pointing to the selected sequence block in the centre of the alignment.

The sequence editor environment (Figure 1) offers a full set of standard editing functions for nucleotide and amino acid sequences, including moving and sorting sequences, entering bases or amino acids manually and searching nucleotide or amino acid sequences or motifs. The display environment is highly flexible, showing nucleotide, split or amino acid sequence representations, a wide range of font styles and sizes, reverse-complement view and different methods for alignment numbering (alignment position or based on a reference sequence, nucleotide or amino acid sequence numbering). A context sensitive, descriptive help file is available for the user as are a variety of help bubbles and command descriptions on the status bar as the various controls and menus are navigated.

An important feature of SSE that contributes substantially to the analytical power of the various analysis and manipulation programs is the ability to classify sequences in the alignment into groups, labelled with tag descriptions. A total of four tag sets each containing 27 different tag groups provide the opportunity for multiple sequence classifications based on different independent attributes (such as, for example in the case of enteroviruses, genome region, species, isolation year and host range). As well as enabling selection and editing of sequences in blocks, grouping with tags defines separate datasets in the various analytical programs. For example, many of the segregation and recombination detection methods require the concept of groups in exploring sequence phylogenies (see next section). Sequence group information is stored in the native SSE file format; as well as tag numbers, groups can be given labels and descriptive annotations.

**Figure 2:**
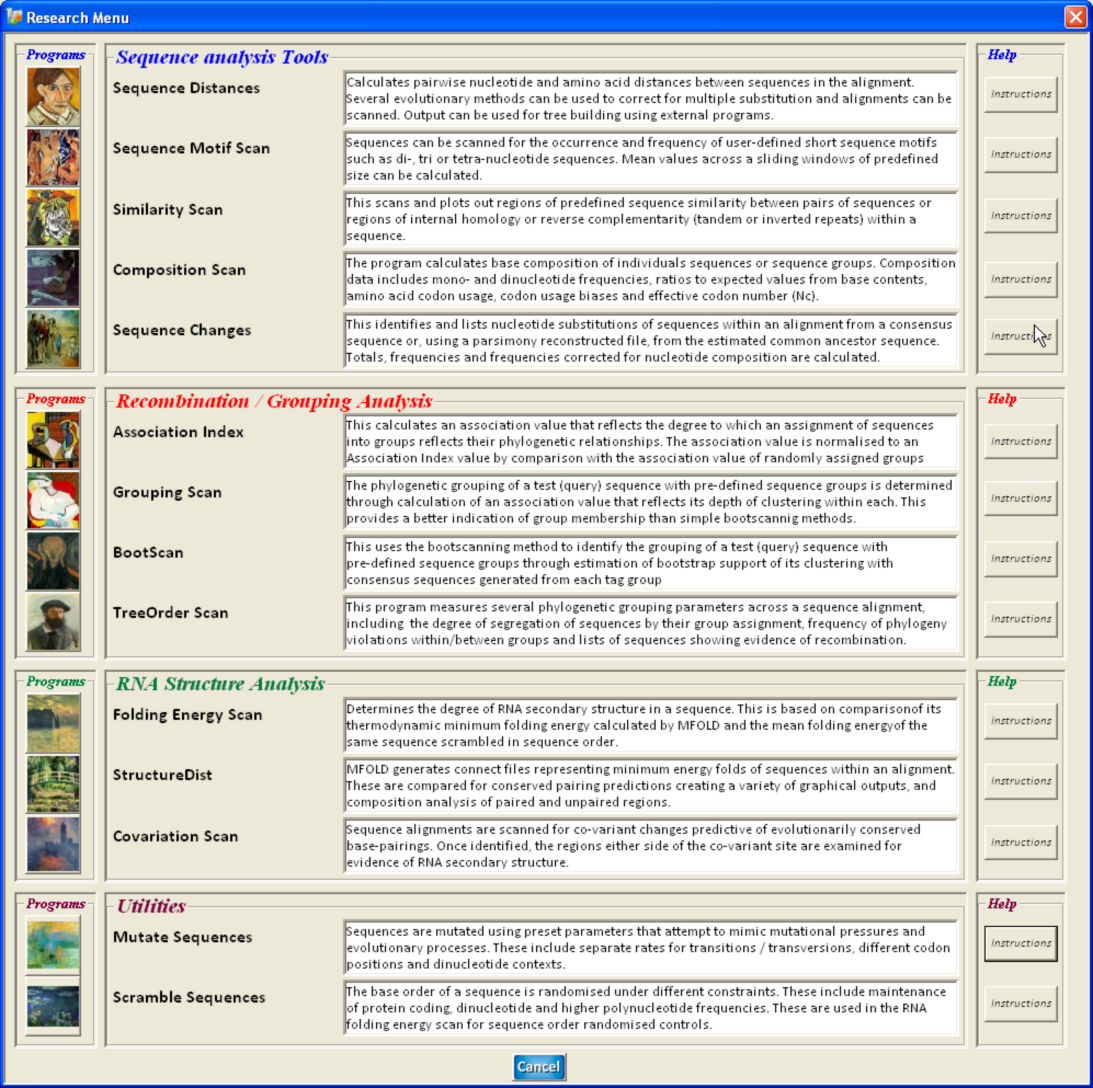
**Research menu options for sequence analysis**. Menu for selection of sequence analysis (divergence, homology, composition), phylogeny (grouping, phylogeny violation detection), structure (RNA secondary structure, covariation) methods and sequence manipulation programs (mutation and sequence order randomization) programs. Program access, command descriptions, brief summaries, summary instructions (buttons on right) and detailed descriptions from a context-sensitive helpfile) are directly available from this menu.

The Research Menu (Figure 2) provides access to a range of newly developed analytical methods and tools for investigation of sequence diversity, composition, phylogenetic grouping and RNA secondary structure that interface directly with sequences in the alignment file. These are described in the following Description section.

The program uses simple ASCII coding for storage of sequence and annotation data in its native format with 4 bit coding for the four bases and each ambiguity code. Alignment and file size is restricted to approximately 4 gigabytes by the 32 bit operating system on which SSE is compiled, although future recompilation into 64 bit code will remove this (theoretical) restriction. In the current version, 2.1 billion sequences of up to 2.1 billion bases in length can be loaded into the editor. However, practical restrictions in loading speed and saving for large datasets arising from the memory handling in Windows limit practical size of datasets to approximately 20-50 MB. The file processes of loading and saving large files are currently being optimised to remove these bottlenecks.

### Description

Apart from acting as a sequence editor and databases for nucleotide and amino acid sequence data and associated annotations, SSE provides built in integration to a series of analysis programs that make effective use of the internal representation of sequences and group assignments for datasets. These are summarised as follows:

### Sequence distances

**Figure 3:**
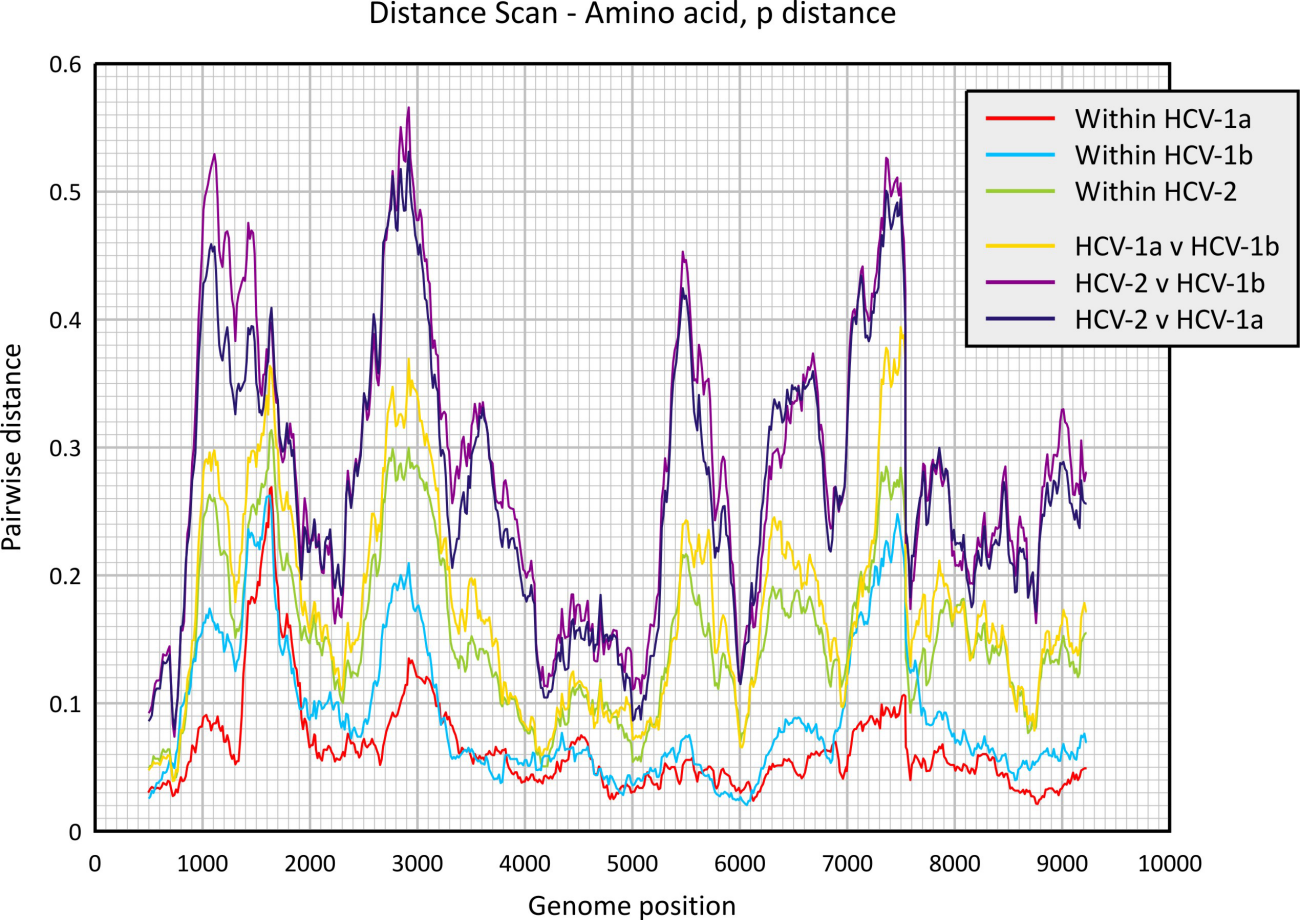
**Program-generated output of sequence divergence scan of uncorrected HCV amino acid p distances**. Program output directly generated from integrated graphics package showing scan of mean translated amino acid sequence divergence between different groups across the HCV genome. Sequences were assigned into three different tag groups based on HCV subtype (15 sequences of HCV-1a, 27 HCV-1b sequences) or genotype (25 HCV-2 sequences).

This program calculates divergence between sequences, as well as scanning alignments scans. Pairwise distances and standard errors are computed between nucleotide and translated amino acid sequences using a variety of evolutionary models. Distances can be separately calculated between individual sequences, mean values calculated between sequence groups or for whole sequence selections. Data output can be optionally directed to distance tables, half-diagonal matrices (PHYLIP or MEGA compatible), tabulated lists for statistical analysis or as graphical output (Figure 3).

Algorithms for distance calculations (uncorrected, Jukes-Cantor, Kimura 2 parameter, Tajima-Nei distances [all sites], synonymous and non-synonymous p, Jukes-Cantor and Li/Pamilo Bianchi distances and for amino acids, p, Kimura and matrix-normalised distances) are based upon distance and standard error formulae presented in [3]. The calculation of matrix distances between translated amino acid sequences uses a standard (PAM) or use-defined similarity matrix.

### Sequence motif scan

This scans alignments for individually defined and entered mono- or polynucleotide motifs, presenting frequencies graphically or output into data tables. Full support is provided for the standard ambiguity codes in motif specification (*eg*. "ACRNU").

### Similarity scan

This scans pairs of nucleotide or translated amino acid sequences within an alignment for regions of similarity, with windows meeting or exceeding the set criteria of number of matches being plotted on a colour coded x/y dot plot graph and/or printed out to a file. Comparisons can be made between or within each sequence in the selected block.

For nucleotide sequence comparisons, fractional scores for sites differing by a transition (*ie*. G and A, C and U/T) rather than a transversion (identical bases score as 1 can be specified. For amino acid sequence comparisons, the standard PAM-Dayhoff matrix can be used to normalise matching scores. It is also possible to specify the reading frames of the two sequences being compared providing a useful method to detect homologous amino acid sequences where the orientation and location of open reading frames in the test sequences is unknown.

### Composition scan

This calculates the nucleotide, dinucleotide and encoded amino acid compositions and codon usage of sequences. Composition values can be generated for individual sequences or averaged over sequence groups for the whole selected alignment. Output is generated in the form of formatted tables or as tabulated lists suitable for spreadsheet import and further analysis.

Dinucleotide frequencies are calculated as frequencies, ratios to expected values based on mononucleotide composition and for coding sequences, normalised for amino acid composition. The latter represent adjusted ratios to expected dinucleotide frequencies that consider amino acid usage of the nucleotide sequence. This correction is based on the observation that the amino acid sequence of an encoded protein predominantly dictates base composition at first and second codon positions, and a proportion of 3rd codon position choices. Non-random choices of various amino acids may lead to skewed mono- and dinucleotide frequencies at each codon position that is untypical of the overall base composition of the sequence. Each dinucleotide frequency can be calculated separately for the three codon positions.

Amino acid composition lists totals of each coding triplet, frequencies of each codon using the total number of codons in the sequence as the denominator, and relative synonymous codon usage (RSCU; [4]). Further output records biases in the usage of individual nucleotides or nucleotide combinations at synonymous positions, creating "Variance" (representing the total scatter of conformity to expected values for each codon) and "Bias" (sum of signed values, therefore reflecting biases in the usage of each base at synonymous positions). Effective codon number (ENc) and associated data are also calculated [5].

### Sequence changes

This enumerates the number of sequence changes of each sequence in a pre-defined group from a reconstructed ancestral sequence. It is used principally to determine substitution patterns of nucleotides between closely related members of a species or other group. Reconstruction of the ancestral sequence is achieved through parsimony reconstruction using the DNAPARS program in the PHYLIP package [6]. Sequence changes between each descendant sequence or node and its immediate ancestor are scored, allowing both the nature of the nucleotide change and its direction to be inferred. Alternatively, a simple consensus sequence can be generated internally, and sequence changes between it and each sequence in the dataset recorded (making the implicit assumption of an absence of tree structure and phylogenetic independence).

Sequence changes to be separately recorded based on their immediate nucleotide context, *ie*. separate lists for sequence changes with different upstream downstream nucleotides (A, C, G and T). This has been included to study, for example, the mutagenic effects of methylation at certain dinucleotide positions (*eg*. CpG). Output data is formatted into tables recording the number and frequencies of sequence identities and changes on analysis of the whole dataset and frequencies of changes only. Separate totals can be obtained for each of the three codons positions.

### Association index (AI)

This program scores the degree of phylogenetic segregation between groups of sequences using a tree scoring method that detects incompatibilities between phylogenetic grouping and group assignment membership (identified as pre-defined tag groups) [7]. The association value *A*, is the sum of the dispersion values from each node, *i *(from a total of *n*) within the phylogenetic tree, representing the degree of heterogeneity of group membership calculated using the following formula:

$$A=\overset{n}{\underset{i=1}{\sum }}1-g_{\text{max}}/2^{t-1}$$

where *t *= number of sequences below that node and g*_max _*is the number of sequences in the most abundant group for that mode. The Association value calculated for native sequences is compared with the nul expectation, *ie*. the score of a tree where group membership is randomly re-assigned to produce the Association Index (*A *of native sequences divided by the mean *A *of group re-assigned trees).

In the SSE implementation, the robustness of tree branches used for AI calculations are evaluated by bootstrap re-sampling of the dataset through nucleotide re-sampling (as implemented in PHYLIP) or internal sequence re-sampling. A Bayesian Markov-Chain Monte Carlo implementation of this method to account for phylogenetic uncertainty has been recently developed [8].

This program originally was used to investigate segregation of variants of human immunodeficiency virus in different anatomical compartments (brain, lymph node, etc.; [7], and between HCV variants infecting injecting drug users in different cities in Scotland and elsewhere [9]. It has since been much more widely used as a population segregation measure in wider biological fields such as ecology.

### GroupingScan

This program adapts the tree scoring method of the AI program to determine group membership of a test sequence to two or more pre-defined sequence groups. Instead of simply recording bootstrap support as implemented in the boot-scanning method [10], the GroupingScan scores how deeply embedded a query sequence lies within each of the clades of each pre-assigned groups (defined by tag groups) and provides a much more robust measure of group membership.

The Grouping Score (*G*) for a predefined group, *a*, can be represented by the following formula:

$$G_{a}=\overset{y}{\underset{i=1}{\sum }}1/2^{N}$$

where *N *= the number of nodes separating the test sequence from each member, *i*, of the group and *y *is the total number of sequences in group *a*. By definition, the total of grouping scores of each pre-assigned group, *G_a _*to *G_n_*,, adds up to one. For robustness, each score is computed as the mean of a pre-defined number of bootstrap replicate trees by nucleotide re-sampling (as implemented in the PHYLIP package) or population re-sampling (selection of a random 2/3rds of sequences from each pre-assigned group). High values of *G *indicate no segregation of the test sequence from the pre-defined group, indicating that it clusters with it.

To localise sites where sequence groupings changed (frequently indicative of recombination), the program automates the analysis of sequential fragments through an alignment, where fragments lengths and step sizes through the alignment can be entered. This program was originally developed for investigation of recombination in hepatitis B virus [11], and a formal comparison with other methods for detecting sites of recombination is planned.

### Bootscanning

This method scan alignments for changes in phylogeny that are indicative of recombination events. It is based on recording bootstrap support for tree constructed from a query sequence and two or more control sequences that are putative recombination parents of the query sequence. The method was first described as a means to identify recombinant HIV-1 sequences [10], and the scanning process automated on the program Bootscan in the Simplot package (http://sray.med.som.jhmi.edu/SCRoftware/simplot/). The version in SSE performs equivalently to the Simplot version, but uses tag assignments, graph and data outputs that correspond in format directly to GroupingScan analysis results.

### TreeOrder scan

This program uses a number of methods to evaluate the relationship between group membership and sequence order in phylogenetic trees generated from their nucleotide sequences. This directly visualises positions in alignments where phylogeny relationship change, for example as a result of recombination. The symmetric difference or partition metric [12] is used to compares the branching orders of phylogenetic trees, and enumerates the number of phylogeny violations between them.

**Figure 4:**
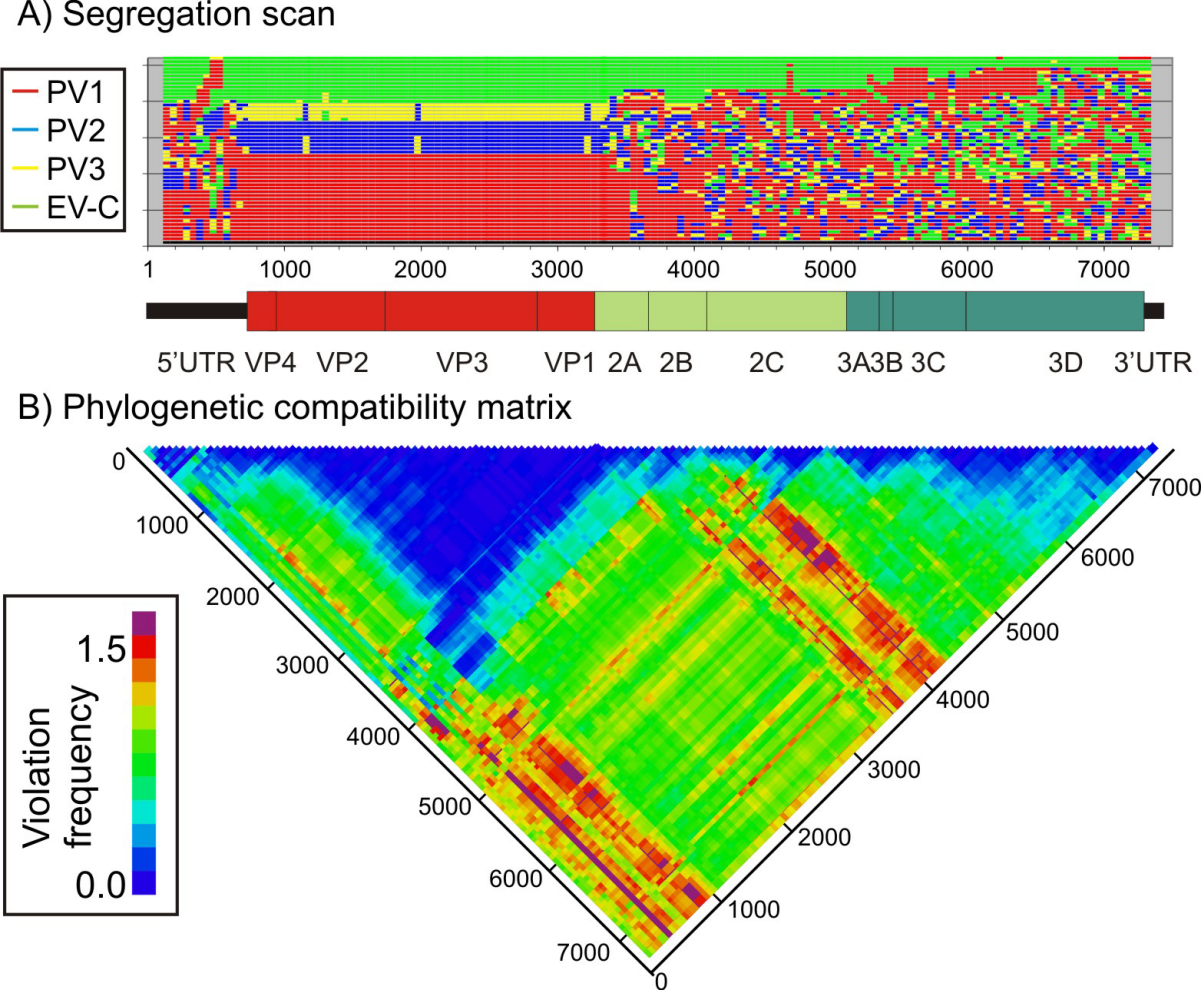
**Ordering of sequence groups and phylogeny violation in different regions of the enterovirus (EV) species c genome**. A) Ordering of sequences from the three poliovirus serotypes (PV1 - PV3) and other enterovirus species serotypes (EV-C) assigned to different tag groups in phylogenetic trees constructed from 250 base sequence fragments sequentially generated across the viral genome alignment. Segregation by serotype occurs only in the VP4-VP1 (capsid encoding region) and is disrupted by recombination events elsewhere [14]. (B) Phylogenetic compatibility matrix of the same dataset show frequency of inter-group phylogeny violations between trees generated from different genome regions.

Program outputs include listings and graphical representation of sequence positions in trees across an alignment and changes in group structure forced by segregation into different, bootstrap supported clades (example shown in Figure 4A). Frequencies of phylogeny violations between trees for a pre-specified bootstrap value can be computed and shown graphically in the form of a half-diagonal matrix using colour coding to represent the degree of phylogenetic incongruence (Figure 4B).

This program has been widely used to study genome positions and constraints on recombination in picornavirus genomes and in other RNA viruses [13], providing an effective visualisation tool for recombination events and gene boundaries (Figure 4).

### Folding energy scan

This program interfaces with UNAFOLD [15] to infer the degree of energetically favoured RNA secondary structure within a sequence or group of sequences. The UNAFOLD algorithm calculates the minimum folding energy (MFE) of a test sequence and compares this value with the MFE of the same sequence scrambled in sequence order. The difference (MFED) represents the sequence-order dependent component of RNA (or DNA) folding. A Z score statistic is computed to record the position (in standard deviation values) of the native sequence within the distribution of control values. MFED values can be computed as a scan across a sequence alignment to localise areas of RNA secondary within a genome.

Although seemingly simple conceptually, the validity of MFED and associated Z-score calculations depends on the method used for sequence order randomisation [16]. Sequence scrambling can use a number of different algorithms that preserve biases in dinucleotide frequencies of the native sequences, codon structure and coding or any combination of these.

### StructureDist

StructureDist compares the most energetically favoured RNA structure predictions for a set of aligned sequences. Minimum energy structures are computed for each selected sequence by UNAFOLD and pairings for each compared to identify conserved and non-conserved structure predictions. The method thus provides a relatively simple means to incorporate information on naturally occurring sequence variability of sequences (with the assumption of underlying conservation of RNA folding) to refine RNA structures prediction. It thus complements stochastic context-free grammar and covariance detection weighting methods that also exploit sequence variability [17,18].

A variety of outputs are produced, recording simple lists of individual base pairings and their frequencies of occurrence and concordance, the structure and sequence contexts in which these pairings occur (position in duplexes, upstream and downstream bases). Other outputs records general conservation of pairing at each nucleotide position and specific pairing predictions that can be represented graphically as matrix graph with colour coding to indicate pairing likelihood.

### Covariance scan

This scans alignments of sequences to determine positions and frequencies of co-variant sites (*ie*. variable self-complementary sites). Following identification, sequences adjacent to covariant sites are scanned for additional base-pairing to verify the existence of duplexed sequence regions. Pairing predictions and text representations of the predicted duplexed regions meeting a range of pre-specified conditions (*eg*. frequency of occurrence, covariant sire variability, pairing conservation) are output in text files.

### Mutate sequences

The program mutates sequences according to sets of specified parameters governing divergence and types of mutation (transitions, transversions, synonymous and non-synonymous). Mutational rates can be additionally specified in different nucleotide contexts; as an example, a much higher rate to C- > T transitions can be specified when followed by a G, mimicking the mutational effect of methylation in mammalian genomic DNA.

### Scramble sequences

The program scrambles sequence order using a variety of algorithms that preserve different naturally occurring attributes of the native sequence. These include biased dinucleotide frequencies, codon structure and frequencies and encoded amino acid sequence. Each of these algorithms is also used in the Folding Energy Scan (see above).

Future developments for this package include recompilation and availability for other operating systems and creation of further display modes for larger sequence datasets created by 454, Solexa and other deep sequencing methods. Algorithms are being generated for *de novo *assembly of larger scale contigs from deep sequencing projects, along with further optimisation of the micro-align method for automated removal of indels and sequencing errors in raw sequence datasets. Further development will additionally be focussed on the phylogeny scanning methods, including incorporation of distance information in the *G *score calculated by the Grouping scan method; the latter program will additionally incorporate boot-scanning as an alternative method to detect changes in phylogeny relationships.

The extremely detailed information generated by the program on the nature and context of sequence changes in naturally occurring viral diversity, combined with detailed analyses of composition biases in nucleotide sequences likely arising through innate immunity-induced selection pressures within the cell will require considerable further analysis and algorithm development in the longer term.

## Conclusions

SSE can be used both as a general purpose sequence editor and organiser of nucleotide and amino acid sequence data on a Windows platform and an analytical tool that includes a variety of diversity, phylogeny and RNA structure algorithms from sequencing and bioinformatic projects. It provides the means to assign sequences to groups, opening up a wide range of more informative methods for analysing sequence variability, phylogeny and RNA structure. The author's research is primarily on viral sequence variation and evolution and program development has primarily followed that focus. However, SSE can also be used for other genetic data, particularly on protein-coding gene sequences where many of the same analytical questions arise.

## Abbreviations

MEGA: Molecular Evolutionary Genetics Analysis; PHYLIP: Phylogeny Inference Package; PAM: Point Accepted Mutation; RSCU: Relative synonymous codon usage; Enc: Effective codon number; AI: Association Index; HCV: Hepatitis C virus; EV: Enterovirus; PV: Poliovirus; MFE: Minimum folding energy; MFED: MFE difference.

## Availability and requirements

Project name: SSE

Project home page: http://www.virus-evolution.org/Downloads/Software/

Operating systems: Windows NT, XP, Vista and 7, Macs running WINE or virtualisation environments (VMware or parallels) with Windows

Programming language: PowerBasic for Windows version 10.03

Licence: Copyrighted to PS, downloadable for personal use on user registration. It should not be re-distributed without permission from author.

Any restrictions to use by non-academics: None

## Competing interests

The author declares that they have no competing interests.

## Authors' contributions

PS wrote the software and the manuscript. All authors read and approved the final manuscript.

## Availability of supporting data

The installation package, copy of licensing terms and any update information are available from the following website:

http://www.virus-evolution.org/Downloads/Software/

This website also allows a standalone formatted program manual (PDF format) to be downloaded without need for program installation.
